# Association between acute methanol poisoning and subsequent mortality: a nationwide study in Taiwan

**DOI:** 10.1186/s12889-018-5918-3

**Published:** 2018-08-07

**Authors:** Jui-Yuan Chung, Chung-Han Ho, Yi-Chen Chen, Jiann-Hwa Chen, Hung-Jung Lin, Jhi-Joung Wang, Chien-Chin Hsu, Chien-Cheng Huang

**Affiliations:** 1 Department of Emergency Medicine, Cathay General Hospital, Taipei, Taiwan; 2 Department of Medical Research, Chi Mei Medical Center, Tainan, Taiwan; 3 Department of Pharmacy, Chia Nan University of Pharmacy and Science, Tainan, Taiwan; 4 Fu Jen Catholic University School of Medicine, Taipei, Taiwan; 5 Department of Emergency Medicine, Chi-Mei Medical Center, 901 Zhonghua Road, Yongkang District, Tainan City, 710 Taiwan; 6 Department of Biotechnology, Southern Taiwan University of Science and Technology, Tainan, Taiwan; 7 Department of Emergency Medicine, Taipei Medical University, Taipei, Taiwan; 8 Department of Environmental and Occupational Health, College of Medicine, National Cheng Kung University, Tainan, Taiwan; 9 Department of Senior Services, Southern Taiwan University of Science and Technology, Tainan, Taiwan; 10 Department of Geriatrics and Gerontology, Chi-Mei Medical Center, Tainan, Taiwan; 11 Department of Occupational Medicine, Chi-Mei Medical Center, Tainan, Taiwan

**Keywords:** Methanol, Poisoning, Intoxication, Mortality

## Abstract

**Background:**

Methanol poisoning (MP) often causes acute mortality and morbidities; however, the association between MP and subsequent mortality has not been well studied.

**Methods:**

We conducted a nationwide population-based cohort study by identifying 621 participants with MP from the Nationwide Poisoning Database and 6210 participants without MP from the Longitudinal Health Insurance Database 2000 by matching the index date at a 1:10 ratio between 1999 and 2012. Comparison of the mortality rate between the two cohorts was performed by following up until 2013.

**Results:**

A total of 249 (40%) participants with MP and 154 (2.5%) participants without MP died during the follow-up (*p* < 0.001). Statistic analysis showed that participants with MP had a higher risk for mortality than did the participants without MP (adjusted hazard ratio [AHR]: 13.48; 95% confidence interval [CI]: 10.76–16.88). The risk of mortality was highest in the first 6 months after MP (AHR: 480.34; 95% CI: 117.55–1962.75). Hypertension, chronic obstructive pulmonary disease, liver disease, malignancy, drug abuse, and lower monthly income also predicted mortality.

**Conclusions:**

MP was associated with increased subsequent mortality. Close follow-up for comorbidity control and socioeconomic assistance are suggested for patients with MP.

## Background

Methanol poisoning (MP) is often caused by volunteer or accidental ingestion. The toxicity is not caused by the methanol itself, but by its metabolite, formic acid [1]. Formic acid will accumulate, resulting in metabolic acidosis and inhibition of cytochrome oxidase in the mitochondria, which lead to histotoxic hypoxia [2]. The brain and the visual pathway are the most sensitive organs to the effect of formic acid, whereas other organs may also be seriously damaged according to the severity of metabolic acidosis [3].

MP causes high mortality and morbidity. Studies of methanol mass poisoning in Estonia, Norway, and Czech have reported 18%–21% of acute mortality due to MP, whereas the sequelae after survival ranged between 10 and 34% [4–6]. Despite an awareness of the toxicity, the incidence of accidental and intentional exposures remains high because it is extremely difficult to distinguish between methanol and ethanol due to the similar characters of color and taste [3–6]. Statistics from the United States showed that nonintentional exposures were reported in 90.3% of all cases, whereas 8.3% were due to intentional exposures [7]. Among the intentional exposures, suicides comprised 51.2% and abuses 38.8% [7].

Majority of studies regarding MP focus on acute mortality and complications; however, knowledge about subsequent mortality is still unclear. When we searched the literature using the key words “methanol,” “poisoning,” “intoxication,” “mortality,” and “long-term,” we found only a small study that recruited 86 patients and reported that MP might increase the long-term mortality. Therefore, to clarify this issue by more and solid evidence, we conducted this nationwide population-based cohort study.

## Methods

### Data sources

The Nationwide Poison Database (NPD) and the Longitudinal Health Insurance Database 2000 (LHID 2000), two sub-datasets of the National Health Insurance Research Database (NHIRD), were used for this study. The NPD contains details of all participants with poisoning recorded between 1999 and 2013 in the NHIRD. The LHID 2000 contains all claims data of 1 million (4.34% of the total population) beneficiaries who were randomly selected in the NHIRD. The NHIRD comes from Taiwan National Health Insurance Program, a universal healthcare system that covers almost 100% of the country’s population [8]. The database of this program contains registration files and original claims data for reimbursement. Large computerized databases derived from this system by the National Health Insurance Administration (the former Bureau of National Health Insurance), Ministry of Health and Welfare (the former Department of Health), Taiwan, and maintained by the National Health Research Institutes, Taiwan, are provided to scientists in Taiwan for research purposes. All the medical expenditures for MP are paid by the National Health Insurance.

### Design

**Fig. 1:**
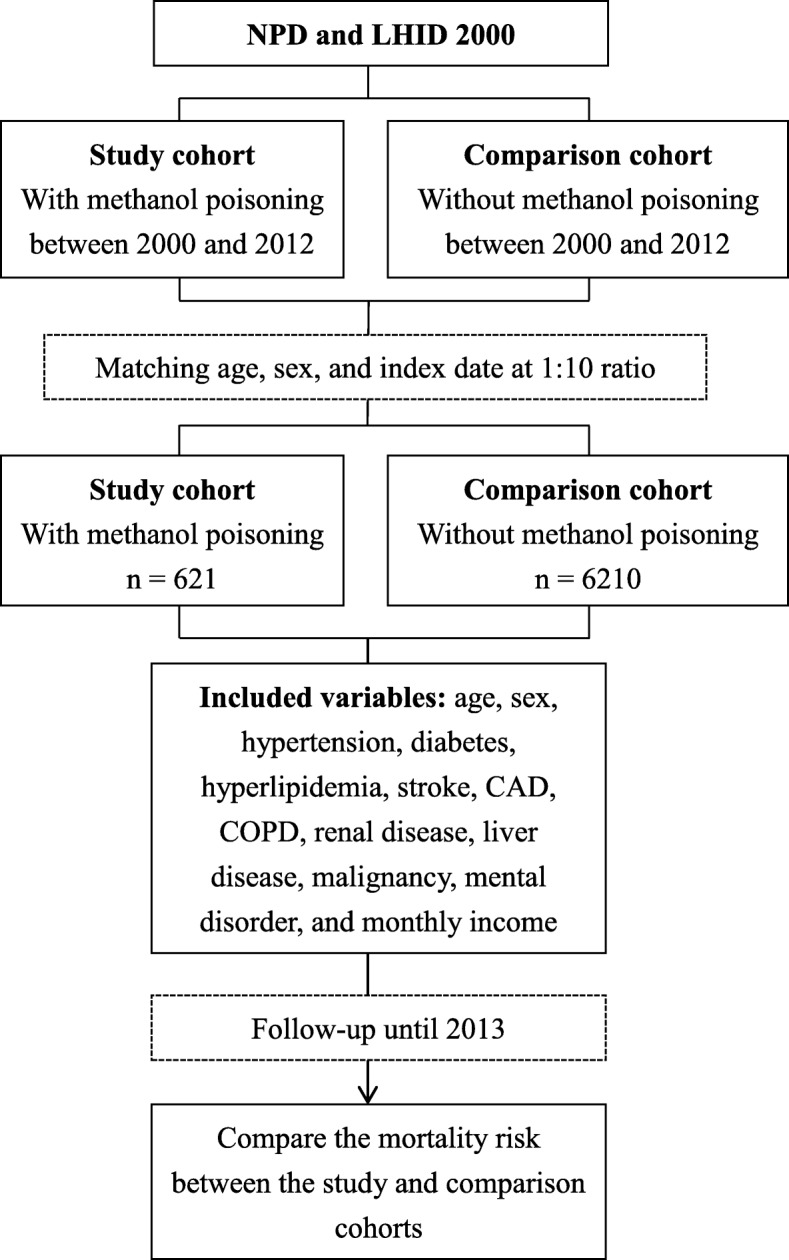
Flowchart of this study. NPD, Nationwide Poison Database; LHID, Longitudinal Health Insurance Database; CAD, coronary artery disease; COPD, chronic obstructive pulmonary disease

We identified participants with MP from the NPD who were newly diagnosed using ICD-9-CM code 9801 or E8602 during admission or emergency department visit between January 1, 1999, and December 31, 2012, as the study cohort (Fig. 1). The diagnosis of MP depended on the treating physicians’ impression after evaluating history of possible exposure, clinical manifestations (e.g., decreased level of consciousness, poor coordination, vomiting, abdominal pain, decreased vision, and a specific smell on the breath), and laboratory data (e.g., acidosis, an increased osmol gap, and methanol level in blood) [3]. The comparison cohort comprised participants without MP who were randomly selected from the LHID 2000 by matching age, sex, and index date at 10:1 ratio with the study cohort. The index date was the date that the participants with MP were first diagnosed.

Comorbidities that may affect the mortality were included if they were diagnosed in more than one hospitalization or in more than three ambulatory cares before the index date and defined as follows: hypertension (ICD-9-CM 401–405), diabetes (ICD-9-CM 250), hyperlipidemia (ICD-9-CM 272), stroke (ICD-9-CM 436–438), coronary artery disease (CAD) (ICD-9-CM 410–414), chronic obstructive pulmonary disease (COPD) (ICD-9-CM 496), renal disease (ICD-9-CM 580–593), liver disease (ICD-9-CM 570–576), malignancy (ICD-9-CM 140–208), drug abuse (ICD-9-CM 303–305), and mental disorder (ICD-9-CM 290–302, 306–319). Monthly income was classified as < 20,000 Taiwan Dollars (TWD), 20,001–39,999 TWD, and ≥ 40,000 TWD [9]. Hemodialysis (HD) was defined as having a management code of 58001C, 58008C, 58027C, 58029C, or 58030B. Acute respiratory failure (ARF) was defined as ICD-9-CM 518.81 or 518.84 or a management code of 960, 960.1, 960.2, 960.3, 960.4, 960.5, 939.0, 939.1, or 311. The ICD-9-CM and management code can be referenced from the websites of the National Health Insurance Administration [10] and the National Health Insurance Research Database [11]. All the participants were followed up from the index date to the date of death or the end of 2013. In Taiwan, all citizens are required to participate in the National Health Insurance, and their enrollment must be withdrawn within 30 days postmortem. Therefore, participants recorded as deceased or disenrolled within 30 days of their discharge were presumed dead, and the discharge date was designated as the date of death.

### Ethics statement

This study was conducted according to the Declaration of Helsinki. The Institutional Review Board at the Chi-Mei Medical Center approved this study and waived the need for informed consents from participants because the dataset consists of de-identified data. This waiver does not affect the rights and welfare of the participants.

### Statistical analysis

We used chi-square test for categorical variables and independent *t*-test for continuous variables to compare the demographic characteristics, comorbidities, and monthly income between the two cohorts. Cox proportional hazard regression analysis by adjusting for hypertension, diabetes, hyperlipidemia, stroke, CAD, COPD, renal disease, liver disease, malignancy, drug abuse, mental disorder, and monthly income was performed to compare the risk of death between the two cohorts. The Kaplan–Meier method with log-rank test for survival analysis was also performed between the two cohorts. We also performed stratified analysis by ARF and HD. Finally, we investigated the independent mortality predictor in all the participants by Cox proportional hazard regression analysis. SAS (version 9.4 for Windows, SAS Institute, Inc., Cary, NC) was used for all the analyses in this study. Significance was set at 0.05 (two-tailed).

## Results

We identified a total of 612 participants with MP and 6120 age-, sex-, and index date-matched participants without MP (Fig. 1 and Table 1). The mean age of both cohorts was 44.39 years (Table 1). The highest percentage of participants were in the age subgroup of 35–49 years (41.71%), followed by 20–34 years (24.32%) and 50–64 years (23.99%). There was a male predominance among participants with MP (82.29%). There were higher prevalences of hypertension, diabetes, hyperlipidemia, CAD, COPD, renal disease, liver disease, drug abuse, and mental disorder and lower monthly income among participants with MP than those in participants without MP.

**Table 1:** Comparison of the demographic characteristics, comorbidities, and monthly income between patients with and without methanol poisoning

Variables	With methanol poisoning *n* = 621	Without methanol poisoning *n* = 6210	*p*-value
Age (years)	44.39 ± 14.02	44.39 ± 14.01	> 0.999
Age subgroup (years)			> 0.999
< 20	9 (1.45)	90 (1.45)	
20–34	151 (24.32)	1510 (24.32)	
35–49	259 (41.71)	2590 (41.71)	
50–64	149 (23.99)	1490 (23.99)	
≥ 65	53 (8.53)	530 (8.53)	
Sex			> 0.999
Female	110 (17.71)	1100 (17.71)	
Male	511 (82.29)	5110 (82.29)	
Comorbidities			
Hypertension	129 (20.77)	942 (15.17)	< 0.001
Diabetes	101 (16.26)	439 (7.07)	< 0.001
Hyperlipidemia	97 (15.62)	548 (8.82)	< 0.001
Stroke	13 (2.09)	75 (1.21)	0.062
CAD	56 (9.02)	376 (6.05)	0.004
COPD	23 (3.70)	99 (1.59)	< 0.001
Renal disease	102 (16.43)	557 (8.97)	< 0.001
Liver disease	270 (43.48)	917 (14.77)	< 0.001
Malignancy	21 (3.38)	179 (2.88)	0.482
Drug abuse	126 (20.29)	80 (1.29)	< 0.001
Mental disorder	210 (33.82)	714 (11.50)	< 0.001
Monthly income ($TWD)			< 0.001
< 20,000	510 (82.13)	3990 (64.25)	
20,001–39,999	81 (13.04)	1454 (23.41)	
≥ 40,000	30 (4.83)	766 (12.33)	

*CAD* coronary artery disease, *COPD* chronic obstructive pulmonary disease, *TWD* Taiwan Dollar

**Fig. 2:**
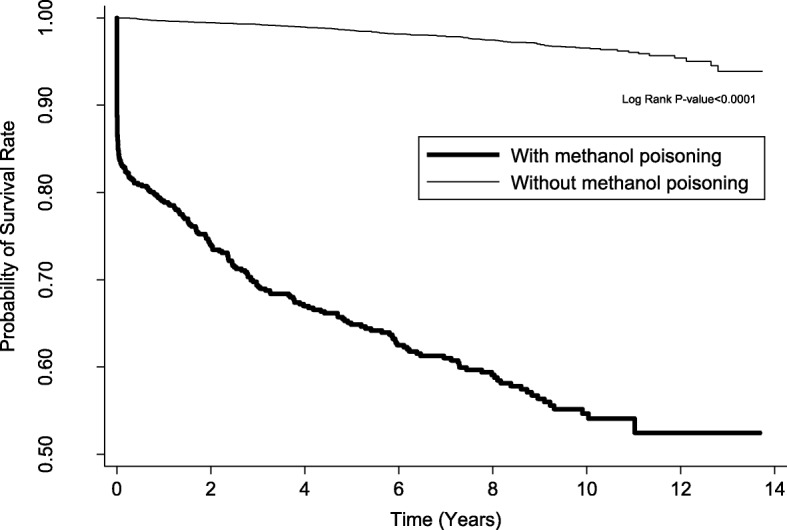
Kaplan–Meier survival analysis with log-rank test between participants with and without methanol poisoning

**Fig. 3:**
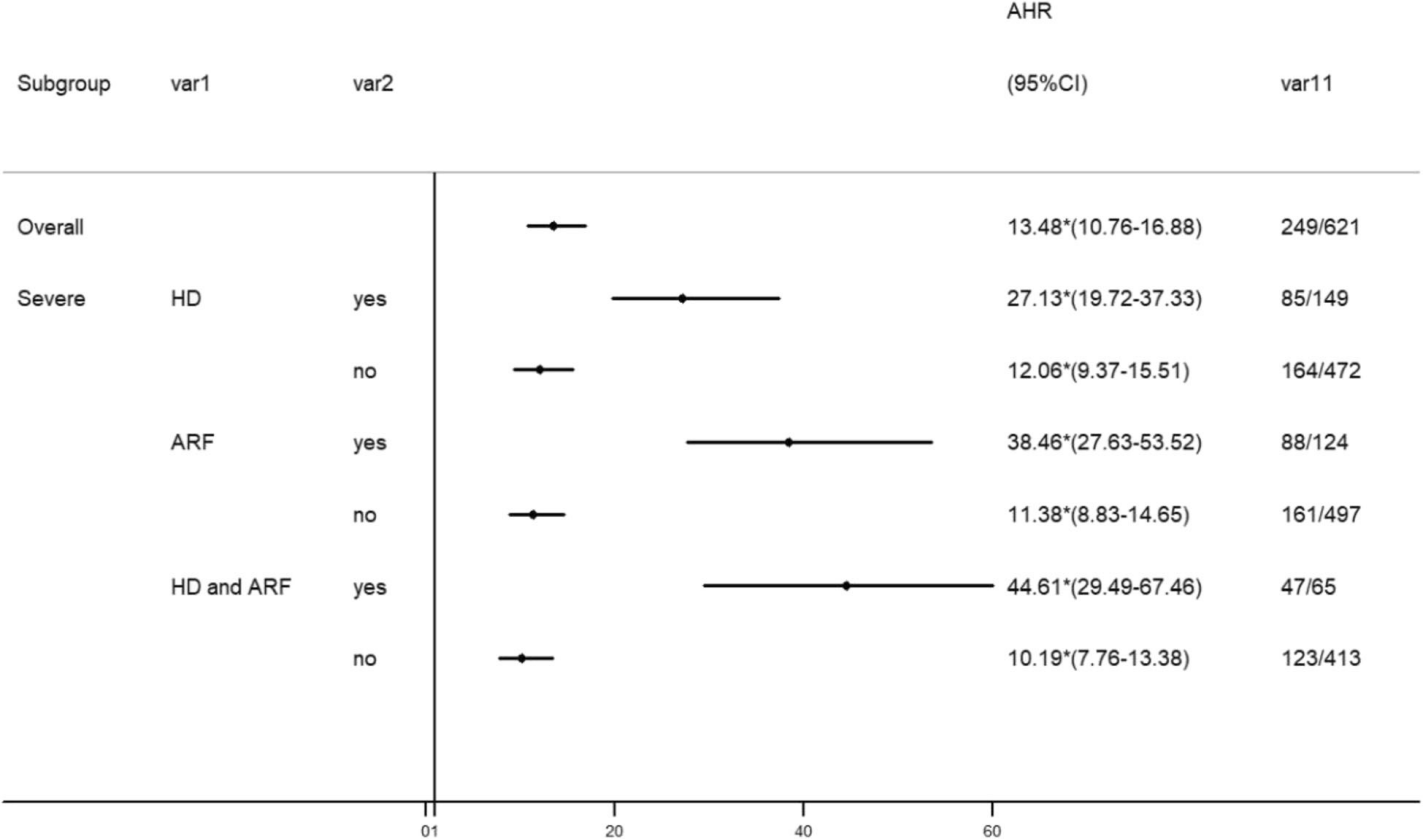
Stratified analysis of subgroups of patients with methanol poisoning. AHR, adjusted hazard ratio; CI, confidence interval; HD, hemodialysis; ARF, acute respiratory failure; CAD, coronary artery disease; COPD, chronic obstructive pulmonary disease. AHR indicated Cox proportional hazard regression analysis by adjusting for hypertension, diabetes, hyperlipidemia, stroke, CAD, COPD, renal disease, liver disease, malignancy, drug abuse, mental disorder, and monthly income

The overall mortality was 40% in participants with MP and 2.5% in participants without MP (Table 2). Cox proportional hazard regression analysis showed that participants with MP had higher risk for death than did the participants without MP by adjusting for hypertension, diabetes, hyperlipidemia, stroke, CAD, COPD, renal disease, liver disease, malignancy, drug abuse, mental disorder, and monthly income (adjusted hazard ratio [AHR]: 13.48; 95% confidence interval [CI]: 10.76–16.88), especially in the age subgroups of 20–34 years (AHR: 31.75; 95% CI: 14.19–71.03) and 35–49 years (AHR: 26.68; 95% CI: 17.25–41.27). There was similar mortality risk in both sexes. The increased risk for death was highest in the first 6 months after MP (AHR: 480.34; 95% CI: 117.55–1962.75) and persistently higher after following up for more than 1 year. We also analyzed the risk for death in the first 2 months after MP, which showed an AHR of 961.21 (95% CI: 133.7–6937.9). Kaplan–Meier survival analysis with log-rank test also showed higher mortality risk in the participants with MP than that in the participants without MP during the follow-up period (Fig. 2). Stratified analyses showed especially higher mortality risk in MP patients with HD (AHR: 27.13; 95% CI: 19.72–37.33), ARF (AHR: 38.46; 95% CI: 27.63–53.52), and both HD and ARF (AHR: 44.61; 95% CI: 29.49–67.46) (Fig. 3). In addition to MP, independent mortality predictors in all the participants were older age, male sex, comorbidity with hypertension, COPD, liver disease, malignancy, and drug abuse and low monthly income by Cox proportional hazard regression analysis (Table 3).

**Table 2:** Comparison of the mortality risk between patients with and without methanol poisoning by Cox proportional hazard regression analysis

Variable	With methanol poisoning n = 621				Without methanol poisoning n = 6210				Crude HR (95% CI)	AHR (95% CI)^a^
	n	Mortality	PY	Rate	n	Mortality	PY	Rate		
Overall	621	249	3105.92	80.17	6210	154	44,990.66	3.42	21.08 (17.21–25.82)	13.48 (10.76–16.88)
Age (years)										
< 20	9	0	76.90	–	90	0	769.01	–	–	–
20–34	151	42	934.42	44.95	1510	9	11,770.88	0.76	53.89 (26.18–110.93)	31.75 (14.19–71.03)
35–49	259	106	1240.12	85.48	2590	33	18,397.97	1.79	41.32 (27.88–61.24)	26.68 (17.25–41.27)
50–64	149	78	659.97	118.19	1490	59	11,022.98	5.35	19.01 (13.50–26.78)	13.10 (8.93–19.25)
≥ 65	53	23	194.52	118.24	530	53	3029.83	17.49	7.24 (4.41–11.88)	8.02 (4.62–13.91)
Sex										
Female	110	27	591.97	45.61	1100	15	7491.75	2.00	20.66 (10.93–39.04)	16.56 (8.12–33.76)
Male	511	222	2513.96	88.31	5110	139	37,498.91	3.71	21.43 (17.30–26.54)	13.42 (10.56–17.06)
Follow-up period										
< 6 months	621	113	85.88	1315.84	6210	1	1031.09	0.97	621.91 (153.64–2517.36)	480.34 (117.55–1962.75)
6–12 months	486	11	237.92	46.23	6071	10	3002.59	3.33	13.85 (5.88–32.62)	6.09 (2.23–16.59)
≥ 1 years	464	113	2617.18	43.18	5943	135	38,911.05	3.47	12.45 (9.70–15.99)	7.70 (5.82–10.21)

*AHR* adjusted hazard ratio, *CI* confidence interval, *PY* person-year, *CAD* coronary artery disease, *COPD* chronic obstructive pulmonary disease

^a^Adjusted for hypertension, diabetes, hyperlipidemia, stroke, CAD, COPD, renal disease, liver disease, malignancy, drug abuse, mental disorder, and monthly income

**Table 3:** Independent mortality predictor for methanol poisoning by Cox proportional hazard regression analysis in all the participants

Variable	Crude HR (95% CI)	AHR (95% CI)^a^
Cohort		
With methanol poisoning	21.08 (17.21–25.82)	13.48 (10.76–16.88)
Without methanol poisoning	1 (reference)	1 (reference)
Comorbidity		
Hypertension	3.36 (2.73–4.13)	1.81 (1.40–2.34)
Diabetes	3.85 (3.02–4.90)	1.23 (0.91–1.65)
Hyperlipidemia	2.29 (1.75–3.00)	0.83 (0.62–1.12)
Stroke	4.23 (2.64–6.79)	1.31 (0.78–2.19)
CAD	2.85 (2.15–3.78)	1.07 (0.78–1.48)
COPD	4.44 (2.96–6.67)	1.71 (1.11–2.61)
Renal disease	2.43 (1.88–3.13)	1.04 (0.79–1.38)
Liver disease	5.74 (4.71–7.00)	2.07 (1.61–2.66)
Malignancy	3.47 (2.43–4.95)	1.82 (1.24–2.69)
Drug abuse	13.21 (10.32–16.91)	1.87 (1.38–2.54)
Mental disorder	4.01 (3.25–4.95)	0.94 (0.72–1.22)
Monthly income ($TWD)		
< 20,000	4.13 (2.46–6.93)	2.30 (1.36–3.88)
20,001–39,999	1.29 (0.71–2.36)	1.13 (0.62–2.07)
≥ 40,000	1 (reference)	1 (reference)

*AHR* adjusted hazard ratio, *CI* confidence interval, *HD* hemodialysis, *ARF* acute respiratory failure, *CAD* coronary artery disease, *COPD* chronic obstructive pulmonary disease, *TWD* Taiwan Dollar

^a^Adjusted for hypertension, diabetes, hyperlipidemia, stroke, CAD, COPD, renal disease, liver disease, malignancy, drug abuse, mental disorder, and monthly income

## Discussion

This study showed that MP was associated with increased subsequent mortality. The mortality rate was especially higher in the age subgroups of 20–49 years, in the first 6 months after MP, and in MP patients with ARF or receiving HD. The majority of participants with MP were males and middle-aged. In addition to MP, Cox proportional hazard regression analysis in all the participants showed that older age, male sex, comorbidity with hypertension, COPD, liver disease, malignancy, and drug abuse and low monthly income were also independent mortality predictors.

One of the possible explanations for the increased subsequent mortality risk in MP is its sequelae, including neurological impairment, renal failure, hepatitis, and visual impairment [6, 12]. MP, similar to ethanol poisoning, may cause depression of central nervous system and subsequent acute respiratory failure in a large dose [1, 2, 12]. The highest mortality in the first 2 months may be due to acute respiratory failure and multi-organ failure via severe metabolic acidosis [1, 2, 12]. Another explanation for the increased mortality risk is that patients with MP have essentially a vulnerable nature to alcohol intoxication and trauma [12]. A 6-year follow-up study about MP reported that the neurological and visual impairments are permanent and were still increasing after the poisoning [12]. Neurological impairment would present parkinsonism-like syndrome, which correlates with computed tomography evidence of destruction in the putamen and subcortical white matter hemorrhage [13]. The patients with sequelae had a mortality rate of 35% during the follow-up, higher than that in patients without sequelae (29%) [12]. The causes of death in the follow-up were alcohol intoxication (27%), cardiac diseases (23%), and traumas (19%), indicating that this is an exposed and vulnerable group [12].

In our study, the majority of patients with MP were middle-aged (35–49 years old) and males, which is compatible with previous studies [5, 12, 14]. A study in Czech recruiting 121 patients with MP reported that the mean age was 54 years (range: 16–79 years) and 80% of the patients were males [5]. Most of the MP cases are related to nonintentional exposure [7, 12]. Middle-aged population is more likely to be addicted to alcohol due to genetic, environmental, and sex hormonal factors [15], and therefore, they might be at higher risk of buying inexpensive and illegal industrial alcoholic beverages, which results in nonintentional MP [12].

Our study showed that although older age predicted mortality, MP had the greatest impact on the mortality of younger participants aged 20–34 years. We did not find any study about this issue in the literature; however, previous studies about other poisonings showed similar findings. A nationwide study reported that carbon monoxide poisoning was associated with increased long-term mortality, especially in the age subgroup of < 30 years, followed by 30–49 years [16]. This phenomenon was also found in another study, which reported that anticholinesterase poisoning increased the long-term mortality, especially in the age subgroup of < 35 years [17]. The authors concluded that the younger population has few comorbidities, and therefore, acute poisonings such as carbon monoxide and anticholinesterase pesticide poisonings may play major roles responsible for their death [16, 17].

Our results showed that MP with ARF was associated with even higher mortality. The probable causes of ARF in MP were altered mental status, hypoventilation, and metabolic acidosis [5]. ARF suggests a higher severity or delayed treatment, which may be the reason for the increased mortality [18].

There were no strict indications for HD in MP, depending on the clinical decision by the treating physician and the medical resources [19, 20]. Despite the ambiguous indication, the American Academy of Clinical Toxicology’s practice guidelines suggest that a methanol concentration of 25 mg/dL is a reasonable indication for HD [21]. Our study showed that patients receiving HD had higher mortality than that in patients not receiving HD, which might be because higher severity is also an indication for HD. Further studies regarding this issue are warranted.

The higher mortality risk in male patients with MP may be related to the activity of alcohol dehydrogenase [22]. Alcohol dehydrogenase oxidizes methanol to formaldehyde, and formaldehyde would further end up into the notorious formic acid, which causes mortality and comorbidity [22]. According to a research on sex-related differences in the hepatic activity of alcohol dehydrogenase isoenzymes, alcohol dehydrogenase activities were significantly higher in males than in females [22]. Liver disease is responsible for the metabolism and elimination of methanol, and therefore, it may also affect mortality [1, 23]. Drug abuse and low monthly income predicted mortality, which may be related to low socioeconomic status and alcohol dependence [14], increasing the chances of buying inexpensive and fake alcohol beverages and delayed seeking of medical assistance.

This study has the strengths of a nationwide design with a large-scale sample size and delineating an unclear issue. Despite these strengths, it has some limitations. First, some variables were not available in this study, including the time between exposure and treatment, causes of poisoning, consciousness level, vital signs, severity of MP, smoking, alcohol consumption, underweight/overweight/obesity, and laboratory data, including blood gas analysis, methanol and alcohol levels, and serum creatinine, which may be related to the prognosis. In order to minimize the effect of possible confounders, we adjusted for COPD, liver disease, drug abuse, hypertension, diabetes, and hyperlipidemia, which could serve as the surrogates for smoking, alcohol consumption, and underweight/overweight/obesity. We also used ARF and HD to stand for higher severity of MP and found that MP patients had especially higher mortality with ARF and/or HD. Second, we did not include the treatments with fomiprazole and alcohol in this study because fomiprazole is rarely available in Taiwan [14] and alcohol is not available in the database we used. Third, we could not differentiate between the causes of death in our study. However, the aim of this study was to investigate the subsequent mortality. Further studies are needed to address this issue. Fourth, participants with MP exposure enrolled in the study might have alcohol drinking habits. Because the data of alcohol consumption is not available, we could not evaluate whether the increased risk of mortality is due to alcohol consumption and distinguish the adverse effect between MP and alcohol. Fifth, it is possible that individuals who had been exposed to methanol (especially low-level exposure) but were not diagnosed as MP were included as controls. Therefore, we could not account for the effect of the potential methanol exposure in these individuals. Sixth, our findings might not be generalizable to other nations due to the differences in treatment, medical resource, and race.

## Conclusions

This retrospective nationwide population-based cohort study showed that there was an association between MP and increased subsequent mortality. The mortality rate was especially higher in the age subgroups of 20–49 years, in the first 6 months after MP, and in MP with ARF and HD. Older age, male sex, comorbidity with hypertension, COPD, liver disease, malignancy, and drug abuse and low monthly income were also independent mortality predictors. Close follow-up for comorbidity control and socioeconomic assistance are suggested for the patients with MP.

## Abbreviations

AHR: Adjusted hazard ratio
ARF: Acute respiratory failure
CAD: Coronary artery disease
CI: Confidence interval
COPD: Chronic obstructive pulmonary disease
HD: Hemodialysis
LHID 2000: Longitudinal Health Insurance Database 2000
MP: Methanol poisoning
NHIRD: National Health Insurance Research Database
NPD: Nationwide Poison Database
TWD: Taiwan Dollars
